# A novel approach of fabricating monodispersed spherical MoSiBTiC particles for additive manufacturing

**DOI:** 10.1038/s41598-021-96187-w

**Published:** 2021-08-16

**Authors:** Zhenxing Zhou, Suxia Guo, Weiwei Zhou, Naoyuki Nomura

**Affiliations:** Department of Materials Processing, Graduate School of Engineering, Tohoku University, Sendai, Miyagi 980-8579 Japan

**Keywords:** Engineering, Materials science

## Abstract

It is very challenging to fabricate spherical refractory material powders for additive manufacturing (AM) because of their high melting points and complex compositions. In this study, a novel technique, freeze-dry pulsated orifice ejection method (FD-POEM), was developed to fabricate spherical MoSiBTiC particles without a melting process. Elemental nanopowders were dispersed in water to prepare a high-concentration slurry, which was subsequently extruded from an orifice by diaphragm vibration and frozen instantly in liquid nitrogen. After a freeze-drying process, spherical composite particles with arbitrary composition ratios were obtained. The FD-POEM particles had a narrow size range and uniform elemental distribution. Mesh structures were formed within the FD-POEM particles, which was attributed to the sublimation of ice crystals. Furthermore, owing to their spherical morphology, the FD-POEM particles had a low avalanche angle of 42.6°, exhibiting good flowability. Consequently, the combination of FD-POEM and additive manufacturing has great potential for developing complex refractory components used in industrial applications.

## Introduction

Metallic alloys with superior mechanical and functional properties at elevated temperatures are in high demand in the aerospace industry^1,2^. Much attention has been paid to refractory intermetallic compounds in academic and industrial fields^2,3^. In the past decade, a particle-strengthened system of the MoSiBTiC type has been developed with a composition 65Mo-5Si-10B-10Ti-10C (at%) via arc-melting technique^4,5^. This alloy had high creep strength at high temperatures^6^, good room-temperature fracture toughness [≥ 15 MPa(m)^1/2^]^7^, and similar or even lower density compared to that of Ni-based superalloys (~ 8.8 g cm^−3^)^8,9^. The MoSiBTiC alloys are considered as attractive structural materials in aerospace applications, owing to their high melting points and superior high-temperature mechanical properties^10,11^. However, it is difficult to manufacture refractory components with complex internal or external structures using traditional methods. Therefore, additive manufacturing (AM) is deemed as a possible alternative^12^.

Laser powder bed fusion (L-PBF) is one of newly developed AM techniques^13,14^. It utilizes a high-energy laser beam to selectively melt metallic powders to build three-dimensional objects in a layer-by-layer model^15,16^. Many factors influence the quality of the L-PBF fabricated parts. Among them, the properties of powders, such as their shape, size distribution, surface morphology, flowability, composition, and laser absorptivity, play a significant role. They directly influence the state of the melting pools, thereby affecting the quality of the final parts^17,18^. Presently, the powders, including Ti-^16,19^, Fe-^20^, Al-^21,22^ and Co-alloys^23^, used for L-PBF, are mostly fabricated by mechanical milling, gas atomization (GA), plasma atomization (PA), plasma rotating electrode process (PREP), and plasma spheroidization (PS)^24,25^. However, each of these methods has significant limitations in the preparation of refractory alloy powders. For instance, powders produced by mechanical milling always have some shortcomings, such as irregular shape, poor flowability, uncontrolled particle size, or contamination^26^. Zhou et al. fabricated MoSiBTiC powders for L-PBF via high-energy ball milling and sieving^12,27^. Although the powder size of ~ 10–45 μm was controlled, abundant internal defects such as cracks were formed in the L-PBF builds, possibly owing to poor flowability of powders and severe contamination. In contrast, GA and PA methods have been widely applied for spherical powder fabrication. Typical particle sizes of GA and PA powders range from 10 to 300 μm^24^. These particles are further sieved to obtain a more narrow distribution. Unfortunately, these methods have limited powder yield, and are time and energy consuming. Moreover, Mo-Si-B alloys cannot be readily subjected to gas atomization due to their high melting points^9^, hence, there have been limited studies on the fabrication of spherical powders of the Mo-Si-B alloys^28^. Recently, PS is a new technique that can be used for refractory powder fabrication^29,30^. The experimental results from Higashi et al.^30^ showed that the PS MoSiBTiC alloy powders had a spherical shape similar to the atomized ones, while the content of elemental Ti or Si was significantly reduced owing to the ultra-high-temperature exposure. Notably, the GA, PA, PREP, and PS techniques require high-quality feedstock materials, such as wires, rods, or hydride alloy powders, further increasing the limitations. The powder manufacturing techniques for refractory materials such as MoSiBTiC alloys are still in development and not well established^12^.

Therefore, in order to extend the application of L-PBF to refractory alloys, it is particularly important to develop an innovative approach for powder preparation to realize a spherical shape and stable elemental composition along with high yielding and low cost. In this study, a novel technique, the freeze-dry pulsated orifice ejection method (FD-POEM), was designed to fabricate monodispersed spherical refractory particles. The working mechanism of FD-POEM, the feasibility of fabricating a high-concentration slurry, and the characteristics of the FD-POEM particles were thoroughly investigated. The present work indicates that FD-POEM process could directly fabricate spherical composite particles with arbitrary elemental ratios, dispensing with preparation of wires and rods in conventional methods. Moreover, no melting-process was performed during FD-POEM, which avoids the loss of low melting-point elements. Particularly, FD-POEM has great potential in preparing the refractory alloy or ceramic powders.

## Experimental

### Principle and experimental procedures of FD-POEM

**Figure 1:**
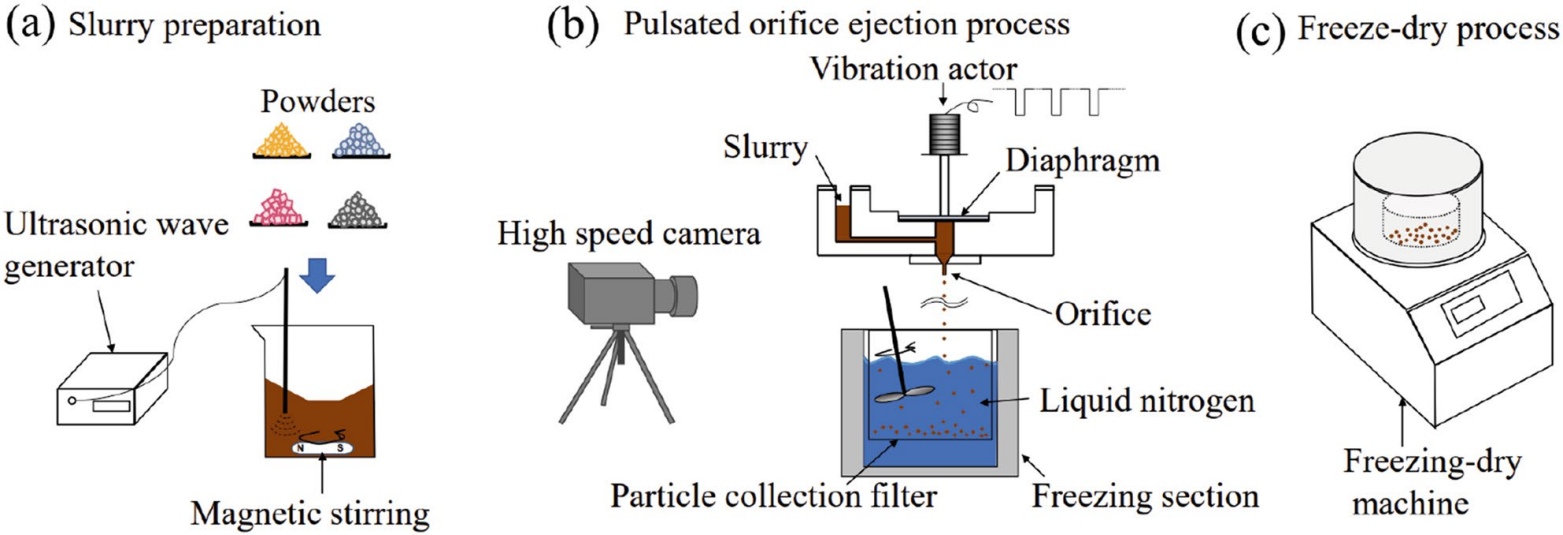
Schematic diagram of FD-POEM process: (**a**) Fabrication process of the slurry; (**b**) Preparation of composite particles by pulsated orifice ejection method; (**c**) Freeze-drying process.

The FD-POEM process involves several steps as follows: (a) slurry preparation (Fig. 1a). Mo, Si, MoB, and TiC submicron powders were selected as elemental materials and weighed according to the nominal atomic composition of 65Mo-5Si-10B-10Ti-10C (at%) alloy. These powders were mixed in a certain amount of deionized water to form a uniform slurry with a concentration of X vol% through combination of mechanical blending and ultrasonication at 273 K for 1 h. Here, X vol% was defined as the volume fraction of powders in the slurry. (b) Pulsed-orifice ejection process (Fig. 1b). The slurry was dropped into liquid nitrogen driven by a pulsated orifice ejection apparatus, which included a pulsated orifice ejection body, diaphragm, and orifice pipe. A diaphragm was used to separate the droplets from the orifice pipe. The outer and inner diameters of the orifice pipe are 1.0 mm and 0.6 mm, respectively. The droplets were extruded from the orifice under diaphragm vibration using a pulse with a frequency of 10 Hz, followed by a square waveform. Owing to the surface tension, the extruded droplets became spherical and then froze instantly when dropped in the liquid nitrogen. (c) Freeze drying process (Fig. 1c). Monodispersed spherical particles were obtained via complete freeze-drying for more than 24 h.

### Characterization

#### Zeta potential and viscosity analysis

The zeta potential of slurries was measured using a zeta potential analyzer (SZ-100, HORIBA, Ltd., JP), which utilizes a laser doppler micro-electrophoresis technique with a red laser wavelength of 633 nm. The viscosity of slurries was evaluated using a viscosity meter (DV2T VISCOMETER, AMETEK BROOKFIELD., US). All zeta potential and viscosity measurements were conducted more than three times. To be consistent with the actual FD-POEM working environment, the zeta potential and viscosity measurements were operated at a temperature of 25 ℃ in this work.

#### High-speed camera observation

Detailed images of the droplet ejection sequence were obtained using a high-speed camera (FASTCAM Mini UX100 type 800 K-M-8G, PHOTRON, INC., JP) with a frame rate of 4000/s and a shutter rate of 160,000/s.

#### Powder feature evaluations

The particle size, distribution, and sphericity of FD-POEM particles were determined using an optical microscope (BX51, OLYMPUS Ltd., JP) equipped with an image analyzer software (WinROOF, MITANI Ltd., JP). The sphericity of FD-POEM particles was determined according to Eq. (1) suggested by the ISO 9276-6 standard^31^.

$${\text{S }} = { 4}\uppi {\text{A }}/{\text{ P}}^{{2}}$$

where S is an indicator for particles' sphericity, P and A are the measured circumference and area covered by a particle projection, respectively. For completely spherical particles, the value of S is equal to 1. As the particle sphericity decreases, the S value will decrease. The particle size, distribution, and sphericity were obtaining by measuring more than 200 particles.

The surface roughness of the FD-POEM particles was determined using a 3D laser scanning microscope (VK-X200 series, KEYENCE Corp., USA).

The flowability of FD-POEM particles were measured using a revolution powder analyzer (Rev2015-Revolution Powder Analyzer, Mercury Scientific Inc., USA) at 25 ℃. The rotation speed was 0.3 rpm. An experimental run consisted of 150 automatically detected avalanche angles. In this work, each avalanche angle measurement was performed more than 3 times.

The microstructure and elemental distributions of FD-POEM particles were evaluated using a scanning electron microscope (JSM-6010LV, JEOL, JP). The internal structure was observed using computer tomography (CT) analyzer (TUX-3200N, MARS TOHKEN SOLUTION Co. Ltd., JP), in which a series of X-ray images was combined to construct cross-sectional morphologies of a FD-POEM powder. The samples were scanned using an X-ray tube voltage of 74.2 kV and tube current of 51.3 μA. A magnification of 45× was used to achieve 1024× 1024 pixels resolution with 1.2 μm pixel size. The exposure time or lamp inclined angle was 2.5 s or 0°, respectively.

## Result and discussion

### Fabrication of uniform slurry

**Figure 2:**
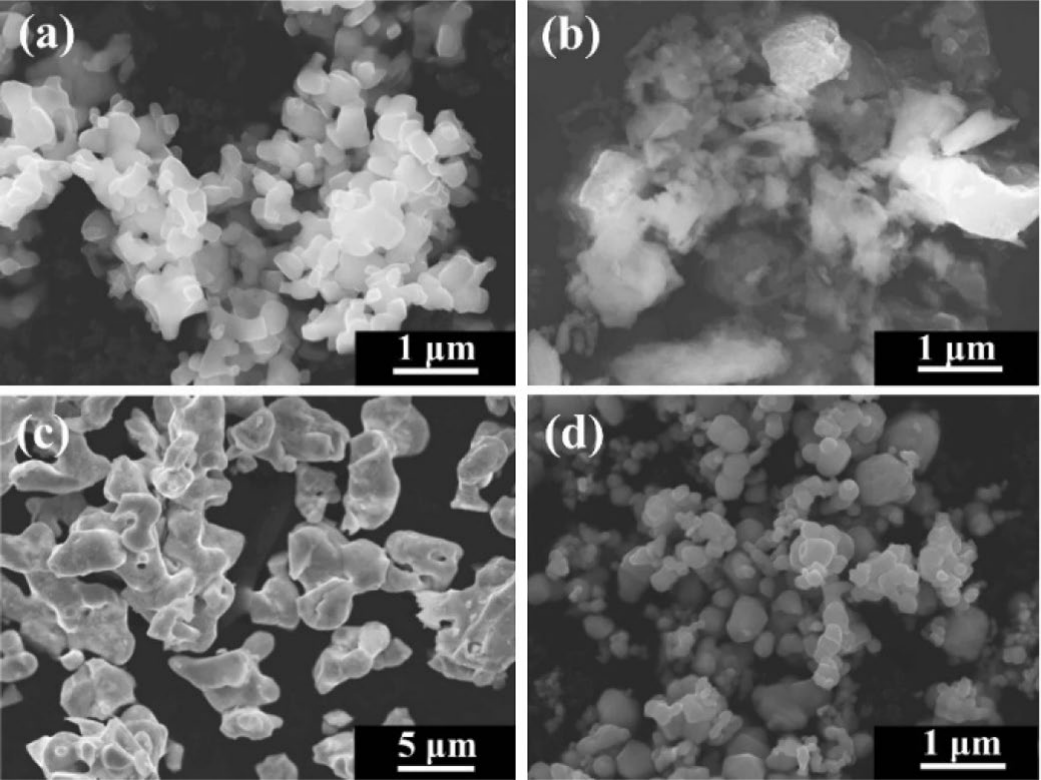
SEM images of the raw (**a**) Mo, (**b**) Si, (**c**) MoB, and (**d**) TiC powders.

The features of the raw materials are deemed to be important for FD-POEM. Figure 2 shows the morphology of the raw powders. It is noted that the particle morphologies and sizes of the raw powders differ greatly, such as polyhedral-shaped Mo and TiC powders (Fig. 2a,d), lamellar-shaped Si powder (Fig. 2b), and irregularly shaped MoB powder (Fig. 2c). According to SEM observations, the medium particle sizes, d_50_, of Mo, Si, MoB, and TiC powders were determined to be ~ 1.0, 4.3, 0.57, and 0.67 μm, respectively.

**Figure 3:**
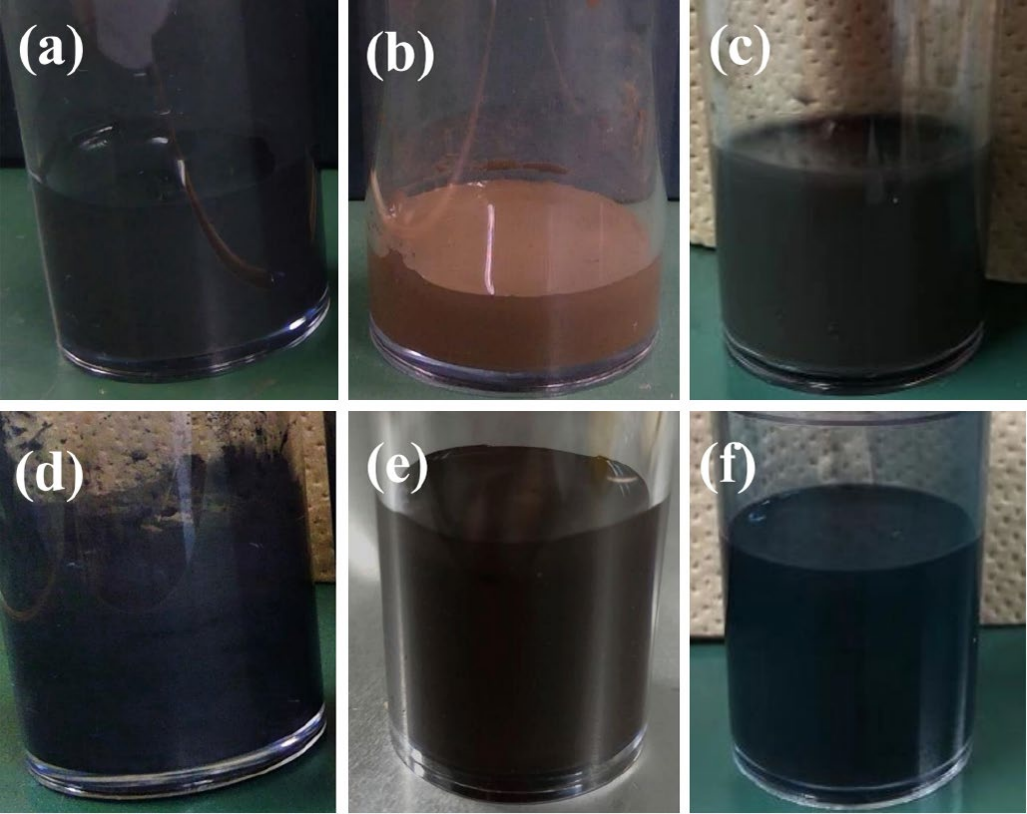
The appearance of 5 vol% (**a**) Mo, (**b**) Si, (**c**) MoB, (**d**) TiC, (**e**) Mo-Si, (**f**) Mo-Si-MoB-TiC slurries.

Single-component slurries of raw Mo, Si, MoB, and TiC powders with a concentration of 5 vol% were prepared separately. Figure 3a–d show the appearance of different single-component slurries. Each slurry had a uniform color without obvious delamination, indicating that the raw powders were well dispersed in water. For comparison, the binary components of Mo-Si and multi-components of Mo-Si-MoB-TiC slurries were also prepared. As shown in Fig. 3e,f, the mixed slurries maintained good dispersion, and the dispersion state did not change with an increase in the amount of the slurry components.

To investigate the stability mechanism of the fabricated slurries several theoretical values that describe the particle motion in water were calculated. The ratio of *d* (Brownian motion distance)*/ν* (sinking velocity) is often applied to characterize the nanopowder stability in slurries. A large *d/ν* value means stable dispersion. In general, the particles can exist stably in water as the value of *d/ν* is larger than 20^32–34^. The sinking velocity of particles follows the Stokes’ law as Eq. (2)^35^,

2 $$\nu =\frac{2{r}^{2}\left(\rho -{\rho }_{0}\right)}{9\eta }$$

where *ν* is the velocity of sinking; $r$ is the diameter of particle; ρ is the density of the particle; ρ_0_ is density of the solution, and η is the viscosity of the solution. The Brownian motion of powders is calculated according to the Fluctuation–dissipation theorem Eq. (3)^35^,

3 $$d=\sqrt{2Dt}$$

where *t* is the time, $d$ is the moving distance of the particle, and *D* is the diffusion coefficient. The diffusion coefficient *D* is expressed as Eq. (4).

4 $$D=\frac{kT}{6\pi \eta r}$$

where *k* is the Boltzmann constant (1.38064852 × 10^–23^ J/K^35^); *T* is the temperature; *η* is the solution viscosity, and *r* is the particle radius. Correspondingly, the *d/ν* values are displayed in Table 1. It indicates that the slurries of Si and TiC are stable in view of the higher value of *d/ν*. However, the relative lower *d/ν* values suggested that the Mo and MoB powders are prone to sink; obviously, this is not consistent with the experimental results shown in Fig. 3.

**Table 1:** The basic parameters of each slurry.

	Mo	Si	MoB	TiC
Viscosity, η (mPa s)	1.03 ± 0.05	1.68 ± 0.05	0.96 ± 0.03	9.66 ± 0.4
Velocity of sinking, ν (nm/s)	2.15	0.08	1.78	0.41
Brownian motion, *d* (nm)	6.75	9.54	6.75	8.24
*d*/ν (s)	3.14	119.25	3.79	20.10

**Figure 4:**
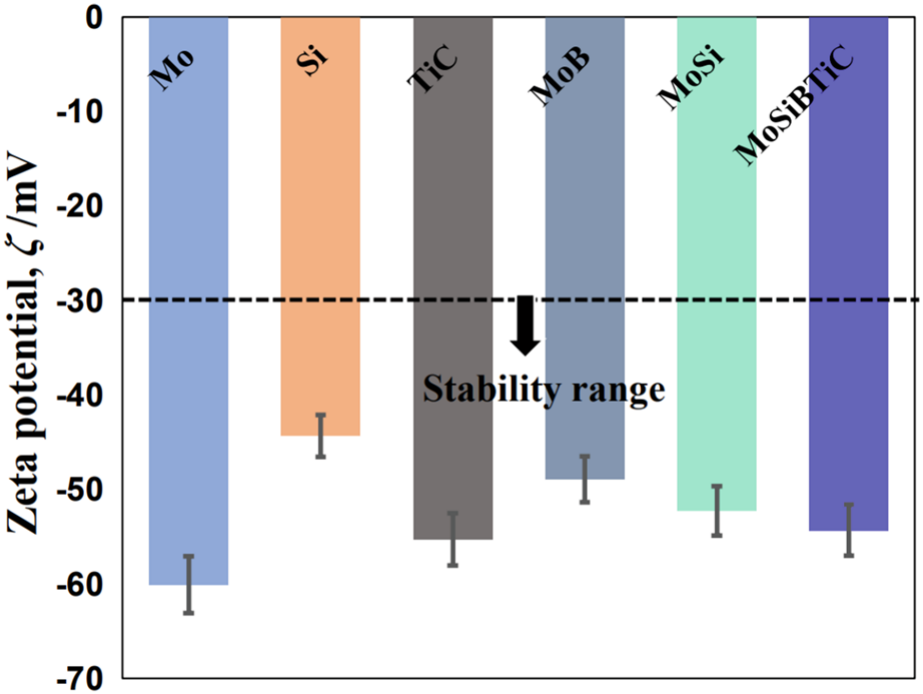
The zeta potential of various powders dispersed in water.

We believe that the discrepancy between the theoretical values and the experimental data shown in Table 1 could be explained by the presence of a repulsive force between particles in the slurry. Herein, the surface electrostatic repulsive force between neighbouring particles is considered, which can be reflected by the zeta potential measurements. It is documented in the literature that when the absolute value of the zeta potential exceeds 30 mV, the particle suspension is in a stable state. As shown in Fig. 4, the absolute zeta potential values of all powders in our experiments were greater than 30 mV, indicating that all powders exerted strong repulsive forces in the slurry. Based on the aforementioned, we concluded that the natural motion and surface charge of powders synergistically contributed to the remarkable stability of the slurries. Because each type of raw powder had the same negative charge, the heterogeneous deposition phenomenon did not occur during the fabrication of the hybrid slurries.

**Figure 5:**
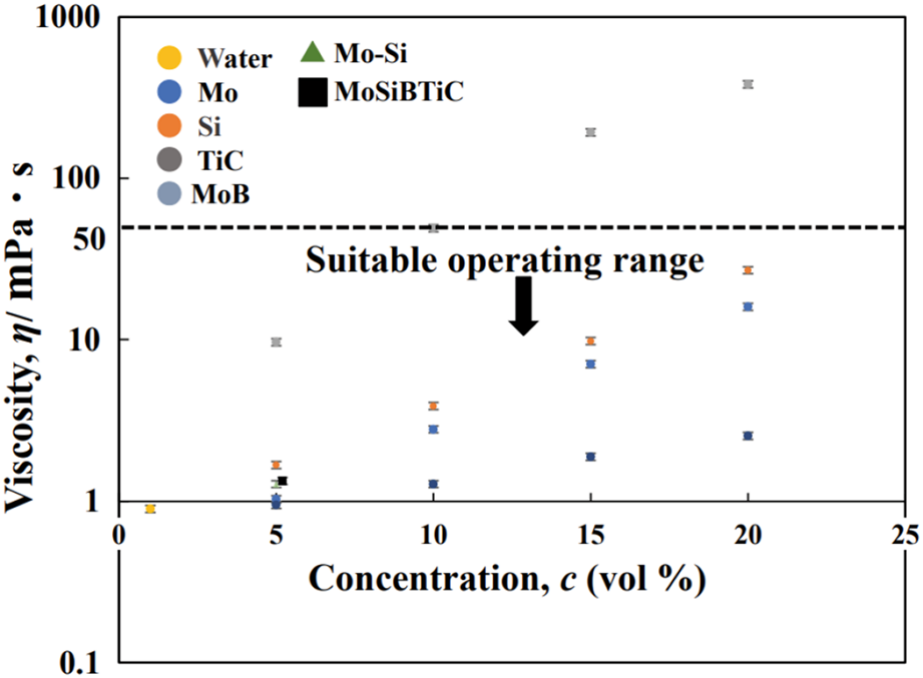
The slurry concentration dependent of the slurry viscosity.

The slurry viscosity is a critical parameter required for the FD-POEM process. Thus, the viscosities of various powder slurries were investigated at room temperature. As shown in Fig. 5, the slurry viscosity exhibits a linear increasing trend with an increase in the slurry concentration. In addition, the range of slurry viscosity that is suitable for FD-POEM was determined. The dotted line in Fig. 5 indicates the viscosity limit of 50 mPa s for the operation. When the viscosity exceeds 50 mPa s, the slurry cannot be ejected from the orifice, regardless of the diaphragm movement.

### Fabrication and evaluation of FD-POEM particles

#### Single-component FD-POEM particles

**Figure 6:**
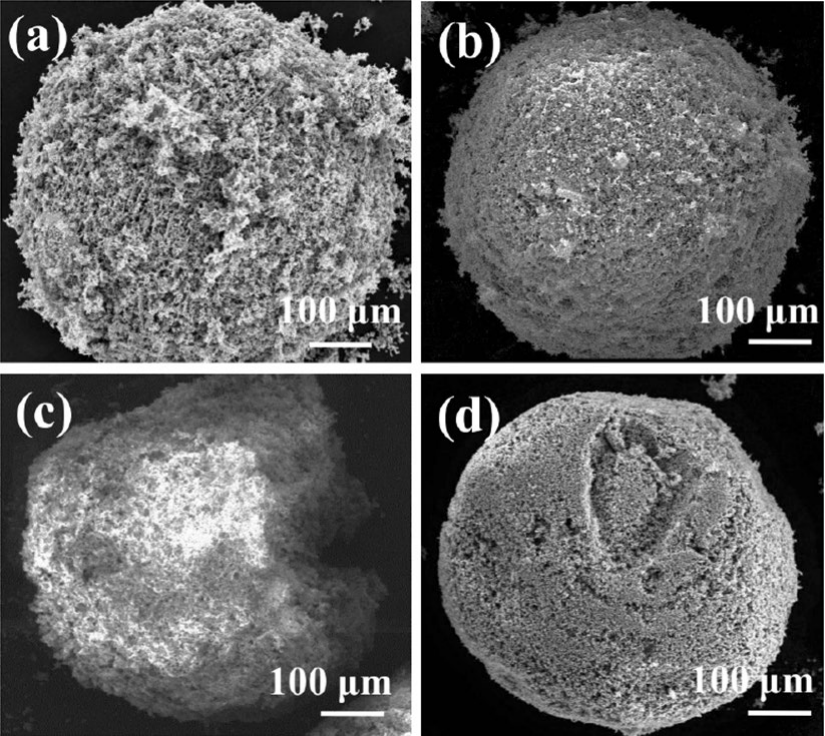
SEM images of the monodispersed single-component (**a**) Mo, (**b**) Si, (**c**) MoB, (**d**) TiC particles by FD-POEM.

Single-component powders of Mo, Si, MoB, and TiC were fabricated individually. The typical morphologies are shown in Fig. 6. As shown in Table 2 and Fig. 6, all FD-POEM particles were nearly spherical with a sphericity value over 0.8, displaying a rough surface. This spherical shaped morphology is beneficial for the powder flow. Many internal pores were present in the FD-POEM particles. The formation mechanism of pores is closely related to the nature of FD-POEM, which will be discussed in “Formation mechanism of pores in FD-POEM particles” section. The surface features of the various FD-POEM particles exhibit significant differences. As shown in Table 2, the Mo and MoB FD-POEM particles exhibited higher surfaces roughness (Ra) than that of Si and TiC particles, agreeing with the SEM observations in Fig. 6. This difference was possibly caused by the size difference of the raw powders. These FD-POEM particles are composed of raw nano or micro-sized particles. First, the small nanoparticles are prone to rearrange and stack owing to the surface tension created during the pulsated orifice ejection process. Second, the interaction between small particles is greater than that of large particles, according to the negative correlation between the van der Waals force and the size of the particles^36,37^. The Si and TiC FD-POEM particles consisting of smaller raw powders exhibited stronger cohesion forces compared to those of Mo and MoB, thereby decreasing the surface roughness.

**Table 2:** The surface roughness (Ra) and sphericity values of different FD-POEM particles.

	Mo	Si	MoB	TiC	Mo-10%Si	Mo-30%Si	Mo-50%Si	Mo-70%Si	Mo-Si-MoB-TiC
Surface roughness (*Ra*, μm)	28.3 ± 5.1	6.4 ± 1.5	36.1 ± 6.7	4.4 ± 1.7	10.8 ± 2.2	7.7 ± 1.2	3.5 ± 2.0	2.3 ± 0.7	5.6 ± 1.8
Sphericity	0.86 ± 0.02	0.93 ± 0.01	0.81 ± 0.02	0.88 ± 0.01	0.90 ± 0.01	0.95 ± 0.01	0.94 ± 0.01	0.94 ± 0.01	0.94 ± 0.01

#### Binary-component FD-POEM particles

**Figure 7:**
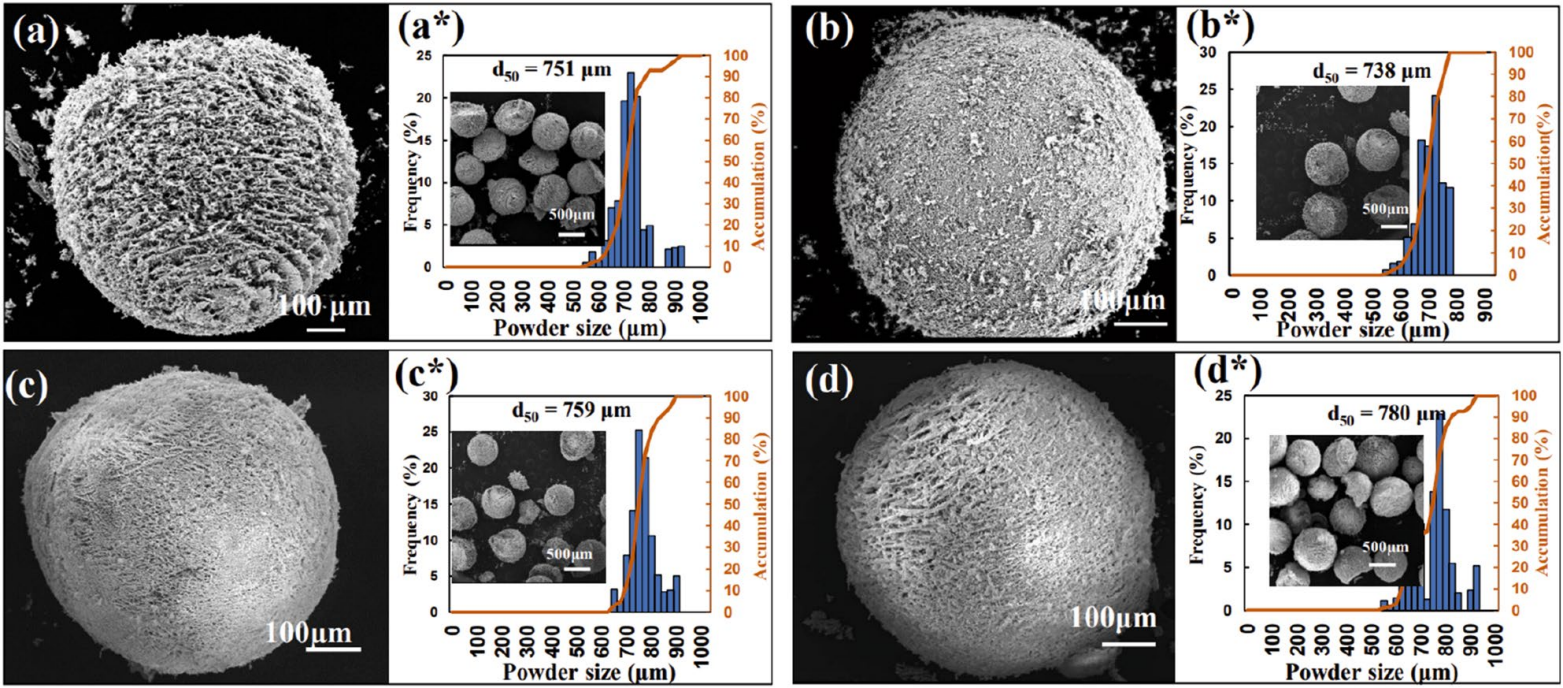
The SEM images and size distribution of the FD-POEM particles (**a**: Mo-10at%Si; **b**: Mo-30at%Si; **c**: Mo-50at%Si; **d**: Mo-70at%Si). The inserts were low-magnification microphotographs.

To verify the feasibility of preparing arbitrary-component particles by FD-POEM, Mo-Si composite particles were fabricated with various elemental ratios. Mo-10at%Si, Mo-30at%Si, Mo-50at%Si and Mo-70at%Si composite slurries with a concentration of 5 vol% were prepared and processed by FD-POEM, respectively. As shown in Fig. 7, as the Si content increased, the surface of the FD-POEM particles became denser and smoother. Correspondingly, the Ra values decreased from 10.8 to 2.3 μm (Table 2). However, the sizes of the Mo-Si FD-POEM particles, d_50_, are similar, in the range of 730–780 μm. This indicates that the composition and concentration of the slurry have an insignificant influence on the particle size of the FD-POEM particles. Therefore, spherical FD-POEM particles with arbitrary composition ratios can be prepared by FD-POEM.

#### Multicomponent FD-POEM particles

**Figure 8:**
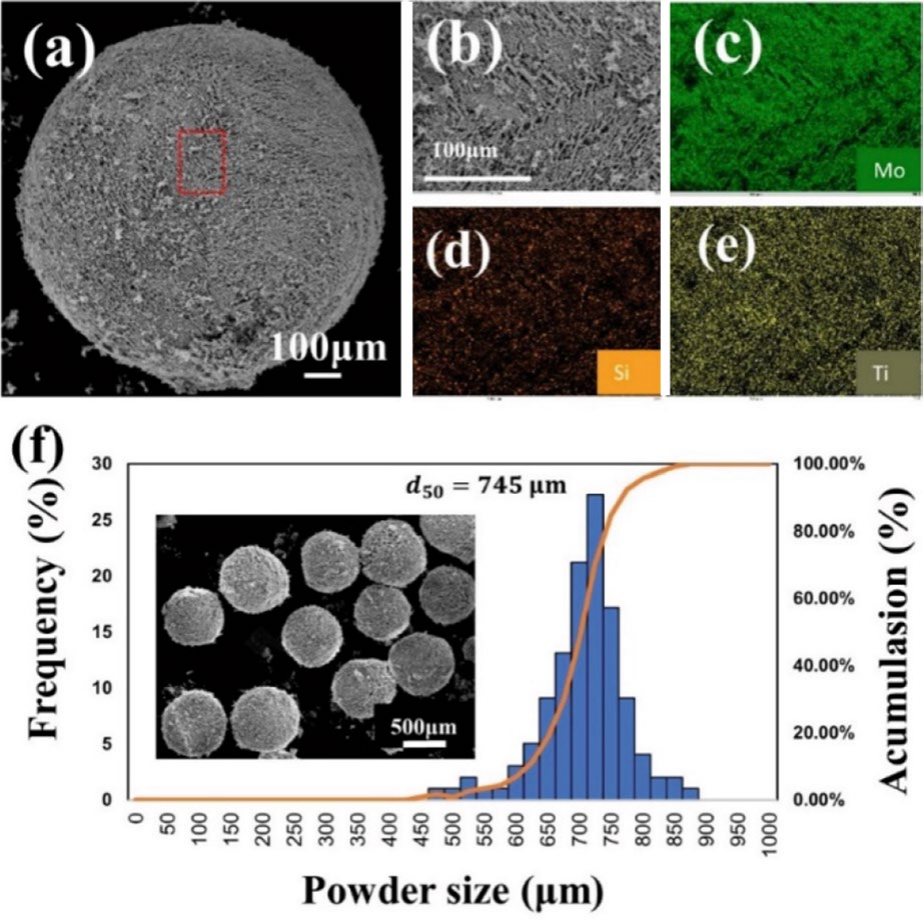
(**a**) SEM images of Mo-Si-MoB-TiC particles by FD-POEM; (**b**–**e**) the enlarged area marked in red box in (**a**) and corresponding EDS mappings; (**f**) the size distribution and low-magnification SEM image of Mo-Si-MoB-TiC particles.

A spherical multi-component particle of MoSiMoTiC was also prepared (see Fig. 8a). As confirmed by SEM–EDS mappings (see Fig. 8b–e), elemental Mo, Si, and Ti were evenly distributed on the particle surface. Figure 8f shows the particle size distribution of the MoSiBTiC particles fabricated by FD-POEM. It can be seen that the size distribution is within a narrow range and follows a typical Gaussian curve. The particle size d_50_ was determined to be 745 μm.

In addition, the powder flowability of FD-POEM particles was reflected by a revolution method, where lower avalanche angles indicated better flowability. The avalanche angle of the FD-POEM MoSiBTiC particles was measured to be 42.6 ± 3°, which is lower than that of the ball-milled alloy powders (~ 50.5°). This flowability improvement was attributed to an excellent sphericity (~ 0.94) of FD-POEM particles.

Notably, this is the first time that refractory composite particles were fabricated directly without a melting process.

### Formation mechanism of pores in FD-POEM particles

**Figure 9:**
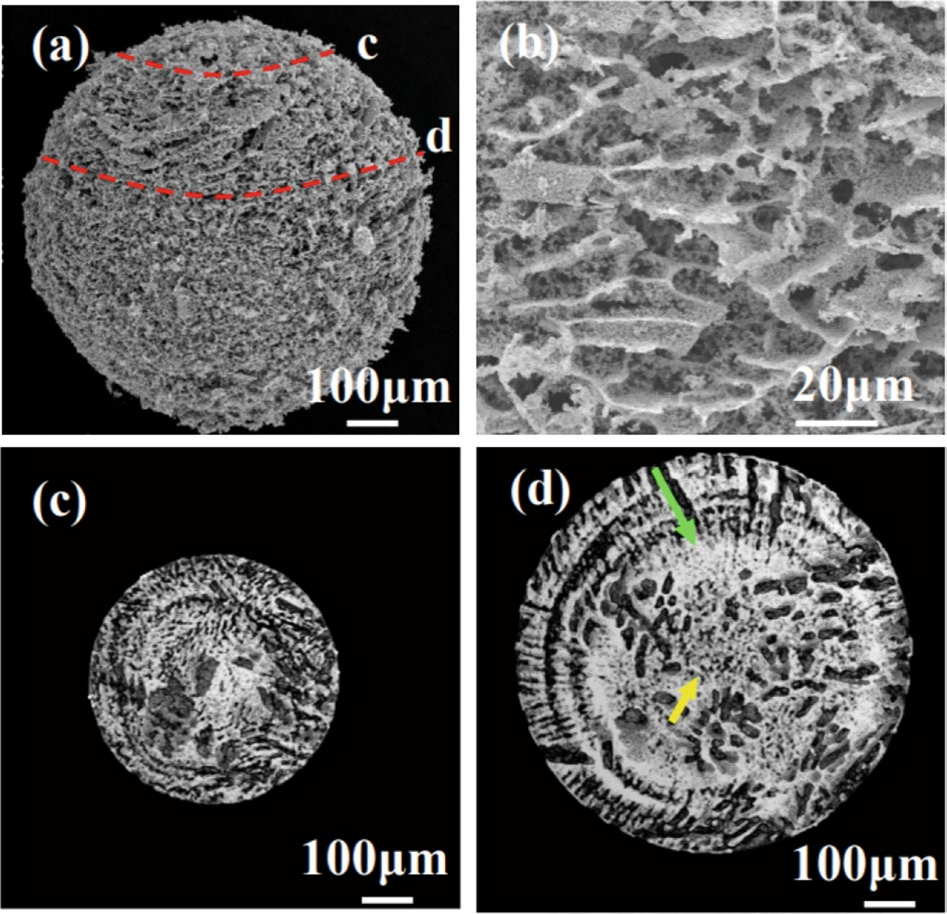
(**a**) low-magnification and (**b**) high-magnification SEM images of a Mo-30at%Si FD-POEM particle. (**c**,**d**) The X-ray CT images of the areas marked by red lines in (**a**).

**Figure 10:**
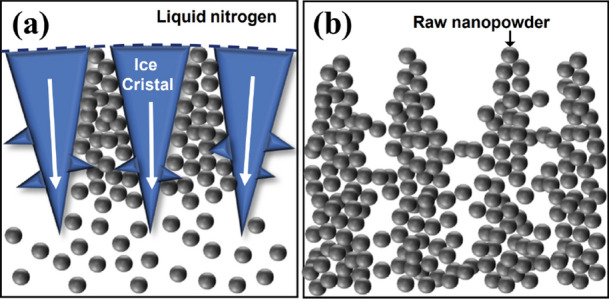
(**a**) Schematic diagrams of (**a**) ice crystal growth during the freezing process and (**b**) ice sublimation during the freeze-drying process. The white arrows in (**a**) indicate the direction of ice crystal growth.

To investigate the internal structure of the FD-POEM particles, we performed high-magnification observations using a field emission scanning electron microscope (FE-SEM). Figure 9a,b show low and high magnifications, respectively of an FD-POEM Mo-30at%Si particle. This particle showed porous structures with flaky topography. The porous feature was further confirmed by cross-sectional X-ray CT as shown in Fig. 9c,d taken from the red line in Fig. 9a. It is noted that the micro-sized voids with radial dendrites (green arrow in Fig. 9d) extend from the outside to the inside. Moreover, some smaller voids were observed in the central area of the particle (yellow arrow in Fig. 9d). These voids were formed during the freezing and drying processes. Once the slurry encounters liquid nitrogen, ice crystallization starts driven by a temperature gradient. The raw powders were rejected by the ice crystal growth and concentrated between the ice crystals, as illustrated in Fig. 10a.

The distribution and movement of nanopowders in the ice crystals were influenced by thermodynamics during crystallization resulting in the formation of voids. Subsequently, the ice crystals containing nanoparticles were exposed to the continuous cooling. The pores were generated further from the sublimation of the ice crystals, leading to the radial distribution of voids from the outside to the inside of the particle (Fig. 10b). Therefore, the spherical FD-POEM particles exhibited a mesh-pored structure following the track of the sublimated ice crystals.

In general, the porosity *p* is a parameter used to evaluate the quality of particles. Here, the porosity value of the FD-POEM particles can be calculated based on Eq. (5):

5 $${{p}} = 1 - \frac{\frac{{\omega }_{1}\times m}{{\rho }_{1}}+\frac{{\omega }_{2}\times m}{{\rho }_{2}}+\dots +\frac{{\omega }_{i}\times m}{{\rho }_{i}}}{V}$$

where ${\omega }_{i}$, *ρ* are the mass fraction and density of component *i*, respectively. *m* and *V* are the weight and volume of an FD-POEM particle, respectively. Correspondingly, the actual porosity was calculated to be 93.2 ± 5 vol%. This value is consistent with the water volume fraction in the 95 vol%. In contrast, the porosity was measured to be ~ 14.0 vol% from the X-ray CT analysis in Fig. 9c,d, which is much lower than the actual value, attributed to the limited resolution of X-ray CT (~ 3.1 µm). This result indicates that ~ 80% of the pores in the PD-POEM particles have a small size (< 3.1 μm). To prove the applicability of FD-POEM particles for L-PBF, a MoSiBTiC build (see Fig. S1 of Supplementary Information) was fabricated successfully using an in-house-developed L-PBF equipment.

In the near future, our main work will focus on the control of FD-POEM particle size by adjusting the pipe orifice size and vibration movement of the diaphragm. The microstructure and mechanical performance of L-PBF parts using FD-POEM particles will also be investigated.

## Conclusion

In this study, a novel powder preparation method, FD-POEM, was systematically developed to fabricate refractory alloy composite particles. The main conclusions are summarized as follows:

1. High-concentration, stable slurries were prepared by dispersing submicron Mo, Si, MoB, and TiC powders in water. It was found that the viscosity of the slurry should be lower than 50 mPa s to meet the operating requirements of FD-POEM.
2. Spherical composite particles with arbitrary composition ratios could be fabricated by FD-POEM. The particles had a narrow size range and uniform elemental distribution. Moreover, many mesh pores ~ 93.2% in volume were formed within the FD-POEM fabricated particles, which was attributed to the ice crystal freezing process and subsequent ice sublimation.
3. Owing to their spherical morphology, the FD-POEM particles had a low avalanche angle of 42.6°, exhibiting good flowability. This study offers new insights into the production of complex system refractory particles, such as MoSiBTiC alloy particles and high-entropy alloy particles for L-PBF.

## Supplementary Information

Supplementary Information.
