# Supplementary material for: A novel approach of fabricating monodispersed spherical MoSiBTiC particles for additive manufacturing
Source: Sci Rep. 2021 Aug 16;11:16576. doi: 10.1038/s41598-021-96187-w (PMC8367977; doi:10.1038/s41598-021-96187-w)
Supplement: Supplementary file 1 — Supplementary Information. [file 41598_2021_96187_MOESM1_ESM.docx]

**Supplementary information**

# **A Novel Approach of Fabricating Monodispersed Spherical MoSiBTiC Particles for Additive Manufacturing**

Zhenxing Zhou, Suxia Guo, Weiwei Zhou* & Naoyoki Nomura*

*Department of Materials Processing, Graduate School of Engineering, Tohoku University, Sendai, Miyagi, 980-8579, Japan*

**L-PBF processing of MoSiBTiC FD-POEM particles**

The detailed L-PBF parameters were as follows: laser power (P) of 20.6 W, layer thickness (t) of 25 μm, hatch distance (h) of 100 μm, and scanning speed (v) of 10 mm·s^−1^. During the entire building process, an argon atmosphere with an oxygen content of < 0.1% was maintained to avoid the degradation of powders and builds.

As shown in Figure S1, a MoSiBTiC build with dimensions of 4 × 4 × 2 mm^3^ was successfully fabricated on the Mo substrate.


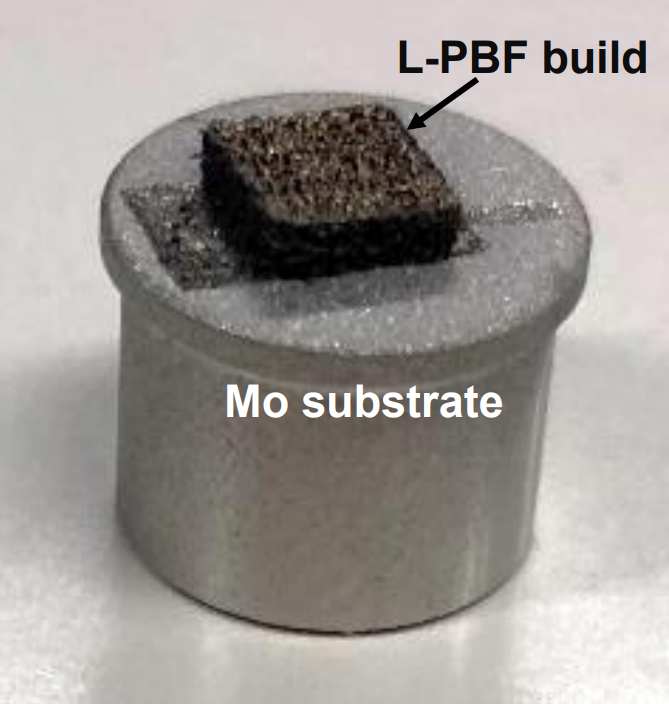


Figure S1. Appearance of a MoSiBTiC alloy build (4 × 4 × 2 mm^3^) on Mo substrate after L-PBF processing of FD-POEM powders.
